# Medium-chain dicarboxylic acids: chemistry, pharmacological properties, and applications in modern pharmaceutical and cosmetics industries

**DOI:** 10.1039/d4ra02598a

**Published:** 2024-05-28

**Authors:** Zhengrui Liao, Yu-Kee Yeoh, Thaigarajan Parumasivam, Wee Yin Koh, Mohammad Alrosan, Muhammad H. Alu'datt, Thuan-Chew Tan

**Affiliations:** a Food Technology Division, School of Industrial Technology, Universiti Sains Malaysia 11800 USM Penang Malaysia 670058674@qq.com; b School of Housing, Building and Planning, Universiti Sains Malaysia 11800 USM Penang Malaysia; c School of Pharmaceutical Sciences, Universiti Sains Malaysia 11800 USM Penang Malaysia thaigarp@usm.my; d Faculty of Food Science and Nutrition, Universiti Malaysia Sabah Jalan UMS 88400 Kota Kinabalu Sabah Malaysia; e College of Health Sciences, QU Health, Qatar University P.O. Box 2713 Doha Qatar; f Department of Food Science & Nutrition, College of Life Sciences, Kuwait University P.O. Box. 5969 Safat 13060 Kuwait; g Renewable Biomass Transformation Cluster, School of Industrial Technology, Universiti Sains Malaysia 11800 USM Penang Malaysia thuanchew@usm.my +604-653 6375 +604-653 6217; h Applied Science Research Center, Applied Science Private University Al-Arab St. 21 Amman 11931 Jordan

## Abstract

Succinic (SUA), glutaric (GLA), pimelic (PA), suberic (SUBA), adipic (ADA), azelaic (AZA), and sebacic acids (SA) make up the majority of medium-chain dicarboxylic acids (MCDAs) with chain lengths of C4–C10, and are widely utilised in the chemical, food, textile, pesticide, pharmaceutical, and liquid crystal sectors. The MCDAs' two carboxyl groups provide them with an incredibly broad variety of applications. The focus of significant scientific research now is on the increasingly varied pharmacological effects of MCDAs. However, only a few studies have compared the biological characteristics of MCDAs in the contemporary pharmaceutical and cosmetic sectors and thoroughly examined the most recent research and marketing initiatives for MCDAs. This review's objective is to offer a thorough analysis of academic works on MCDAs, to assess the usefulness of these substances' chemical–pharmacological properties for use in the contemporary pharmaceutical and cosmetic industries, and to investigate the direction of their possible applications in these two disciplines. In addition, this review investigates how these compounds are metabolised in the human body.

## Abstract 2

Diverse chemistry, pharmacological properties, and applications in the modern pharmaceutical and cosmetics industries were reported and discussed for 7 medium-chain dicarboxylic acids.

## Graphical abstract

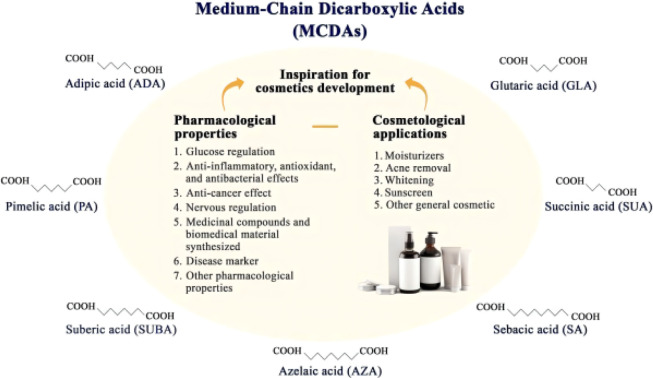

## 1. Introduction

Medium-chain dicarboxylic acids (MCDAs) with chain lengths of C4–C10, which are widely used in the chemical, food, textile, pesticide, pharmaceutical, and liquid crystal industries, primarily consist of succinic (SUA), glutaric (GLA), adipic (ADA), pimelic (PA), suberic (SUBA), azelaic (AZA), and sebacic acids (SA).^1^ Due to their safety and other characteristics, numerous dicarboxylic acids (DAs) and their salts have been reported to be used directly or unintentionally as food additives. The two carboxyl groups in the MCDAs give them an extraordinarily wide range of applications.^2^ But it is important to note that pharmacology and cosmetics may undervalue their beneficial biological effects.

Over the past 15 years, important scientific studies, including patents, have concentrated on the increasingly diverse pharmacological effects of MCDAs. For instance, research on glucose regulation, anti-inflammatory, antioxidant, antibacterial, antivirus, anti-cancer, and other pharmacological properties are included in them.^2–5^ Yet, their efficacy applications in the cosmetic industry are very limited, mainly including moisture, acne removal (including blackheads), (hyper)pigmentation removal (whitening), and sun protection.^6–8^ In terms of cosmetic efficacy, moisturising and acne removal are directly or indirectly related to anti-inflammatory, antioxidant, and antibacterial properties.^9^

Consequently, as a valuable class of substances, MCDAs are important in the pharmaceutical and cosmetic industries, and their pharmacological properties are prospective for the development of efficacy in cosmetic products.^10^ However, few reports have correlated the biological properties of MCDAs in the modern pharmaceutical and cosmetic industries and analysed in depth the latest research and promotional activities related to MCDAs.

The goal of this review is to provide a systematic review of scholarly publications on MCDAs. To compare the relevance of the chemical–pharmacological properties of SA, SUA, AZA, GLA, ADA, SUBA, and PA for applications in the modern pharmaceutical and cosmetic industries to explore the direction of their potential applications in these two fields. In addition, the metabolic processes of these substances in the human body are investigated in this review.

## 2. The MCDAs

**Fig. 1:**
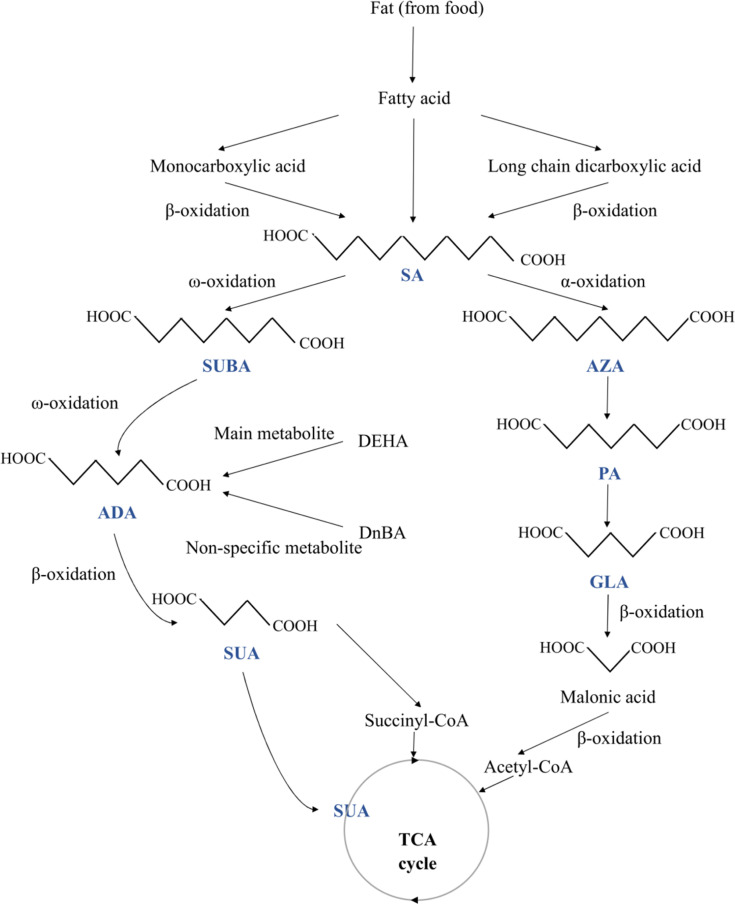
The metabolic relationship between succinic (SUA), glutaric (GLA), adipic (ADA), pimelic (PA), suberic (SUBA), azelaic (AZA), and sebacic (SA) acids. Abbreviation information: adipic acid ester di(2-ethylhexyl) adipate (DEHA); di-*n*-butyl adipate (DnBA); tricarboxylic acid cycle (TCA cycle).

The physical characteristics of the MCDAs differ greatly, which may be related to their chemical structure (Fig. 1). The members of the shorter chain (*e.g.* SUA and GLA) are crystalline solids that are only moderately soluble in organic solvents and highly soluble in water. Water solubility dramatically reduces across the ADA to SA chain lengths, *e.g.* ADA is non-hygroscopic, yet they remain soluble in hot water.^1,11^ In other words, the length of these acids' chains affects how soluble they are in water. When the carbon number shifts from even to odd, there is a noticeable fluctuation in melting point. GLA is an example of odd members that have lower melting temperatures and greater solubility than even carbon number MCDAs, *e.g.* SUA and ADA. These alternating effects are thought to be caused by odd carbon number compounds' incapacity to assume both carboxyl groups are oriented in-plane concerning the hydrocarbon chain.^1^ In fact, the odd members have more energy than the even ones. The dissolving process releases this energy differential, which reduces the solution's heat. Therefore, the increased solubility of odd members can be attributed to stretched torsional conformations. But to twist the molecular backbone, the acids need energy, which dissolves the crystals and releases them. Since molecular torsion energies are significantly larger than van der Waal interactions, which are more prevalent for long chain lengths, it is to be expected that they predominate for compounds with short chains.^12,13^

While many MCDAs are found in natural sources (Table 1), historically, these acids have been produced for use in the industry by pyrolyzing lipids with alkali.^2^ The authors revealed that the molecular structures of all MCDAs are similar, but PA is most likely an anthropogenic product, whereas SUBA and AZA most likely come from fatty acids.^14^ According to Hyder *et al.*,^14^ PA is created when SUBA and AZA are continuously oxidised down to lower-carbon numbered acids. Apart from PA, SUA, a citric acid cycle intermediary, is present in practically all plant and animal cells but in very small amounts.^2^ Conversely, AZA is a dicarboxylic acid (DA) that occurs naturally and can be obtained through food sources like whole grains and animal products. Endogenous sources include oleic acid metabolism, β-oxidation of monocarboxylic acids, and longer-chain DAs. AZA's endogenous plasma levels and daily urine excretion are very reliant on food intake.^15^ Likewise, castor, ironweed (*Vernonia galamensis*), and Rose of Sharon (*Hibiscus syriacus*) are just a few of the plants that contain large amounts of SUBA. SUBA is produced both endogenously in the human body and exogenously by plants.^16^ But ADA is not frequently found in nature. It can be found in sugar beet and beet red juice in nature, in contrast to GLA, which is.^16^ Interestingly, SA is rarely ingested because it is almost absent in food commonly consumed but is present in deficient levels of honey.^17^ However, recent studies have found that SA accounts for nearly 22% of the lipid extract of royal jelly.^18^

**Table 1:** Characteristics for succinic (SUA), glutaric (GLA), adipic (ADA), pimelic (PA), suberic (SUBA), azelaic (AZA), and sebacic acids (SA)

MCDAs^a^	Chain length^b^	Water solubility (g/100 g)^c^	Melting point (°C)	Sources	Ref.
SUA	C4	4.72	185	Endogenous fatty acid metabolism, plants, industrial synthesis, and food	2, 12 and 13
GLA	C5	63.41	97.5	Endogenous fatty acid metabolism and industrial synthesis	2, 12 and 13
ADA	C6	2.05	153	Endogenous fatty acid metabolism and industrial synthesis	2, 12, 13 and 16
PA	C7	2.52	105	Endogenous fatty acid metabolism and industrial synthesis	2, 13 and 14
SUBA	C8	0.035	144	Endogenous fatty acid metabolism, plants, industrial synthesis, and food	2, 12–14 and 16
AZA	C9	0.06	106.5	Endogenous fatty acid metabolism industrial synthesis and food	2 and 12–15
SA	C10	0.005	132	Endogenous fatty acid metabolism, plants, industrial synthesis, and food	2, 12, 13, 17 and 18

a Medium chain dicarboxylic acids (MCDAs).

b Number of carbon atoms.

c Solubility in g of solute per 100 g of water at temperature closest to 25 °C taken from values reported in 15 to 25 °C range.^12^

## 3. Metabolic studies

When the β-oxidation of free fatty acids is compromised, DAs are naturally produced metabolic products of the β-oxidation of monocarboxylic acids. MCDAs can also be directly converted by a series of β-oxidations of long-chain DAs.^19^ But, with longer chain lengths, there was a decrease in the number of MCDAs found in plasma (Fig. 1). DAs are rapidly β-oxidised under normal physiological circumstances, resulting in extremely low cellular quantities and virtually undetectable concentrations in the plasma. Peroxisomes and mitochondria both β-oxidize MCDAs. Odd- and even-numbered chains undergo oxidation but at various ends. Acetyl-CoA and malonic acid (C3) are produced *via* the β-oxidation of odd-chain DAs.^2^ At that point, oxidation cannot continue, and malonic acid serves as the precursor to fatty acid production. Even-chain carboxylic acids undergo full oxidation, yielding succinyl-CoA as an intermediate metabolite and a gluconeogenic substrate. DAs will diffuse less readily across typical cell membranes because they are more polar than their esters.^2^ After oral and gavage administration of AZA or SA to 30 groups of male Wistar rats, DA catabolites with 2-, 4-, or 6-fewer carbons than the equivalent DA were found in the plasma.^20^

All tissues showed AZA metabolism, with the liver, lungs, and kidneys having the highest concentrations after 12 hours. All organs' AZA levels after that gradually declined, except adipose tissue, where growing levels were still detectable at 96 hours. AZA was mostly localised in the fatty acid part of triglycerides and phospholipids, where it made up around 90% of the AZA identified in the tissues' lipids. Within the first 24 hours, traces of C9, C5, and C7 DAs were found. Up to 72 hours after dosage with AZA, the GLA and PA were discovered in the urine.^20^

ADA and SUBA are created *via* the ω-oxidation pathway, which is a process that some species of animals, including humans, use to metabolise fatty acids. This pathway is an alternative to β-oxidation, where the carbon (the carbon farthest from the fatty acid carboxyl group) is oxidised instead, a process that primarily takes place in the endoplasmic reticulum. When β-oxidation is damaged or impeded, this process is typically a minor catabolic pathway for medium-chain fatty acids (10–12 carbon atoms)-becomes more significant. The removal of dangerous quantities of free fatty acids may be accomplished through ω-oxidation in some pathophysiological conditions, including diabetes, drunkenness, and hunger. In the mitochondria, β-oxidation occurs, and when mitochondrial metabolism is compromised, β-oxidation may also be disrupted. Puig-Alcaraz *et al.*^21^ hypothesised that changed mitochondrial metabolism can boost omega fatty acid oxidation activity, increasing the generation of ADA and SUBA. SUBA excretion in urine was significantly higher in patients with many conditions than in healthy participants, including medium-chain acyl-CoA dehydrogenase impairment, fatty acid oxidation disorders, and diabetes.^22^

The breakdown of longer aliphatic DAs in the mammalian liver was shown to produce many carboxylic acids, including ADA.^16^ Similar to how fatty acids are metabolised through β-oxidation, ADA also undergoes this process.^23^ By way of β-oxidation, ADA is converted into SUA and acetic acid. ADA is only partially metabolised in humans, though.^24^ It has been demonstrated that in humans, ADA is a significant metabolite of the adipic acid ester di(2-ethylhexyl) adipate. However, di-*n*-butyl adipate's main, non-specific metabolite was ADA. Besides that, acetate is a metabolite of ADA.^20^ Any ADA that is not metabolised is excreted in the urine.^24^

## 4. Application of the pharmacological properties of MCDAs and their safety in use

MCDAs have recently been considered prospective replacement fuel substrates in both healthy and pathological human conditions.^25^ Although MCDAs' relatively high level of urine loss is a drawback (which diminishes as their chain length increases), this does not prevent parenteral nutrition preparations, such as those for decompensated diabetic mellitus, from using MCDAs.^26^ In addition to this, MCDA has other pharmacological properties.

### 4.1 Glucose regulation drugs

**Fig. 2:**
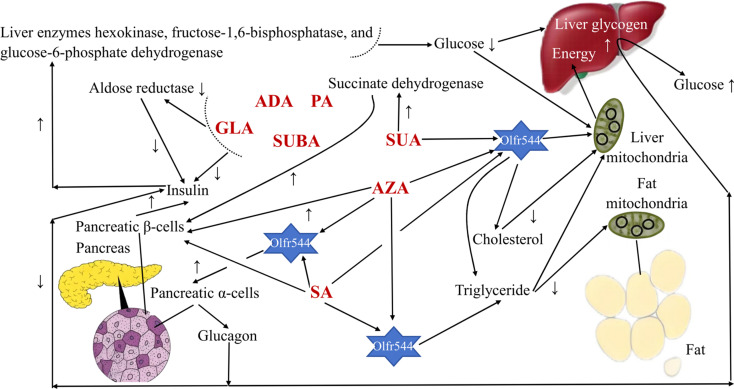
The glucose regulation pathways of medium-chain dicarboxylic acids (MCDAs). Abbreviation information: succinic acid (SUA); glutaric acid (GLA); adipic acid (ADA); pimelic acid (PA); suberic acid (SUBA); azelaic acid (AZA); sebacic acid (SA); olfactory receptor 544 (Olfr544).

In humans, MCDAs can compete with glucose for cellular uptake (Fig. 2). Aldose reductase activity, as well as both phases of glucose-induced insulin release by the perfused rat pancreas preparation, are potently inhibited by GLA and its equivalents.^27^ Directly, the presence of glucose causes the release of insulin from pancreatic β-cells when SUA, SA, and AZA are present.^3,28,29^ AZA therapy significantly increased the amount of hepatic glycogen in type 2 diabetes patients and mice with high-fat diet-induced diabetes. That is, the activity of the liver enzymes hexokinase, fructose-1,6-bisphosphatase, and glucose-6-phosphate dehydrogenase was all boosted, which resulted in more glucose being used for energy production and more liver glycogen, which stabilised blood sugar levels.^30^ Additionally, after a high-fat diet, AZA was able to return the triglyceride level to a close-to-normal range.^30^ Azelates, however, have not been utilised much as medications but have been identified as members of a novel pharmacological class. In actuality, AZA's weak water solubility limits its application.^31^ It has been discovered that SA and AZA can bind to an MCDA receptor known as olfactory receptor 544 (Olfr544). Adipose tissue, the liver, and the gut had the highest levels of functional receptor Olfr544. AZA-driven Olfr544 activation significantly contributes to the promotion of mitochondrial biogenesis by triggering the cyclic adenosine monophosphate-response element binding protein-peroxisome proliferator-activated receptor gamma coactivator 1-alpha signalling axis.^32^ In other words, it has been proposed that Olfr544's ectopic expression in metabolic tissues regulates cellular energy metabolism.^32^ Indeed, Olfr544 is expressed in mouse islet pancreatic α-cells and secretes glucagon in response to AZA and SA.^33^ According to Yamaga *et al.*,^35^ the higher SA content might be advantageous for controlling blood sugar. It has been shown that consuming SA (23 g) as part of a varied meal lowers postprandial glucose and hepatic glucose production in type 2 diabetic patients without increasing insulin secretion.^34^ However, there are still only a few clinical research using oral SA to treat type 2 diabetes, most of which are animal studies.^35^ When SUA is active, the state of the tissues defines the strength and direction of the change, and the outcome is shown by optimising the parameters that influence how well they work. SUA dramatically boosted succinate dehydrogenase activity, which had an insulinotropic impact in experimental diabetes.^36^ In experimental diabetes mellitus brought on by the injection of streptozotocin (65 mg kg^−1^) in rats, SUA (50 mg kg^−1^) decreases blood glucose and cholesterol levels, inhibits lipid peroxide, and treats abnormalities in oxidative phosphorylation in the liver mitochondria.^37^

### 4.2 Anti-inflammatory, antioxidant, and antibacterial drugs

**Fig. 3:**
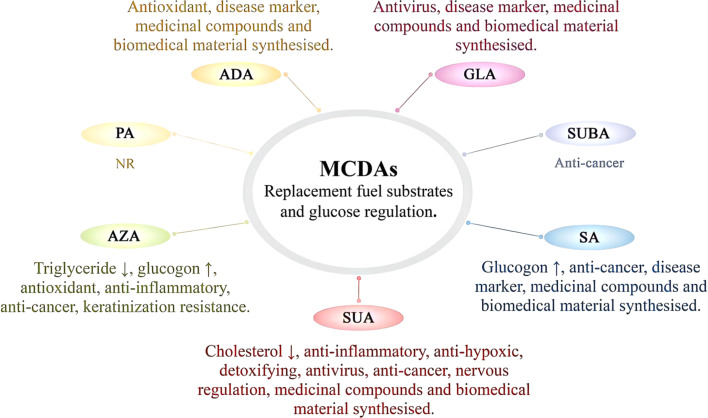
The usage of medium chain dicarboxylic acids' (MCDAs') pharmacological properties. Abbreviation information: succinic acid (SUA); glutaric acid (GLA); adipic acid (ADA); pimelic acid (PA); suberic acid (SUBA); azelaic acid (AZA); sebacic acid (SA); not reported to be used (NR).

In general, all MCDAs may have bacteriostatic properties. Still, in terms of anti-inflammatory and antioxidant properties, the results may vary depending on the type of MCDAs (Fig. 3).^4^ AZA acts as a scavenger of reactive oxygen species, a competitive inhibitor of a variety of oxidoreductive enzymes *in vitro*, and a suppressor of oxyradical toxicity in cell cultures. This suppression also applies to the dangerous hydroxyl radical. Meanwhile, it prevents neutrophils from producing reactive oxygen species, and it may naturally act as an antioxidant in the body.^38^ Some of its most significant general characteristics include the non-toxicity of AZA, similar to SA, and the absence of hazardous metabolite production.^39^ AZA is often used as a bactericidal, anti-inflammatory, and antioxidant agent.^40^ For example, AZA is used topically to treat skin conditions such as cutaneous hyperpigmentation disorders and acne rosacea, a chronic acneiform ailment that affects the skin.^41^ It has antibacterial, anti-inflammatory, and keratinisation-resistance qualities.^42^ Through the inhibition of nuclear transcription factors, which reduces the inflammatory cascade of cytokines, including interleukin-1, interleukin-6, and tumour necrosis factor, studies have shown that AZA has an anti-inflammatory effect. Antibacterial action is connected to the alteration of intracellular pH and metabolism in bacteria.^43^ The generation of reactive oxygen species in healthy keratinocytes was also slightly inhibited by it.^44^

Unlike AZA, whether SA has antioxidant properties has not been specifically reported. In contrast, by regulating numerous proteins connected to nuclear factor kappa-B and mitogen-activated protein kinase (MAPK) signalling, SA displays anti-inflammatory effects.^45,46^ To our knowledge, ADA, along with SA, did not trigger lipid peroxidation or impair antioxidant potential in the brains of young rats *in vitro*, suggesting that the mechanism by which ADA modifies brain function is unknown. However, it does not appear to be related to oxidative stress.^21^

SUA is an endogenous metabolite that is used in a variety of therapeutic procedures.^47^ The researchers investigated the pharmacological effects of many pharmaceutical formulations, including SUA. The use of SUA in paediatrics and the treatment of mitochondrial illnesses is the subject of a study.^47^ It is present in many species and contributes considerably to the biochemical processes of cell respiration in varying levels because all aerobic biosystems use the citrate cycle.^48^ Therefore, SUA affects the mediatory, cellular, and molecular processes that regulate the immune system.^49^ Anti-hypoxic, detoxifying, and antioxidant effects of SUA have been demonstrated.^36^ It was found that SUA has an anti-inflammatory effect on hepatitis and even liver cirrhosis. Gallstone elimination is made easier, salt generation is increased, and liver drainage is aided.^36^ Low-density lipoprotein cholesterol and very low-density lipoprotein cholesterol levels in the blood were consistently reduced after drinking SUA. The atherogenicity index reduced while the high-density lipoprotein cholesterol content increased.^36^ By bringing the body's metabolism back to normal, SUA boosts the immune system. As a result, it is suggested for the clinical therapy of immune deficits and infectious diseases. For instance, Reamberin™ 1.5% solution for infusions is used to prepare SUA for the treatment of intestinal infections, specifically rotavirus gastroenteritis, shigellosis, salmonellosis, klebsiella infection, and dysentery with no known cause.^49^

While not affect the activities of catalase and superoxide dismutase, GLA at concentrations ranging from 0.05 to 2.0 mM dramatically enhanced chemiluminescence (up to 65%) and decreased total radical-antioxidant potential (up to 28%) and glutathione peroxidase activity (up to 46%).^50^ The findings show that GLA causes oxidative stress in the rat brain in a cultured environment. It is probable that these discoveries, if they show up in humans, will alter the neuropathology of glutaric acidemia type I patients.^50^ Notably, GLA, as a virucidal organic acid, is effective at inactivating rhinovirus on human skin.^51^ To inactivate rhinoviruses, the carboxyl functionalities of GLA may bind to or interact with their surface proteins. For maximum virucidal activity, the carboxyl groups might need to be in a specific conformation or spaced at a specific distance apart. The conformation, the distance, or both could be impacted by the alkane bridge's length, steric interactions caused by substitutions on the bridge, or a combination of these factors. There may also be a correlation between substitution at the C-3 position and the degree of protonation of the carboxyl functions since protonation of the carboxyl group increases the virucidal activity of GLA.^52^ Besides that, a method of action independent of an acidic pH and low temperature appears to be used by GLA to inactivate rhinovirus type 14 and several other strains of human rhinoviruses. SUA had roughly the same activity in the test system as GLA despite having one less carbon than GLA.^52^

By contrast, at various concentrations (10^2^–10^3^ M), ADA inhibited the hydroxide anion-induced photo storage chemiluminescence of acridine. Thus, ADA is used in food preservation because of its good antioxidant activity but is less often mentioned in clinical settings.^53^ Normally, ADA has a strong bacteriostatic effect, *e.g. Escherichia coli*.^54^ In this regard, PA is also employed as a building block for antibacterial chemicals.^55^

### 4.3 Anti-cancer drugs

The abnormally hyperactive and cancerous epidermal melanocytes react negatively to MCDAs, which cause cell death (Fig. 3).^2^ MCDAs, C8 to C13, have been demonstrated to reversibly block microsomal NADPH and cytochrome P450 reductase as well as mitochondrial oxidoreductases. *In vitro*, MCDAs are also active tyrosinase competitors.^2^ AZA is currently recognised to have antitumor effects on a variety of diseased cells, including cutaneous malignant melanoma and human choroidal melanoma. AZA is a promising anti-cancer drug that dose-dependently triggers apoptosis and dramatically reduces the viability of acute myeloid leukaemia cells.^38^ It may prevent the creation and generation of cellular energy in acute myeloid leukaemia, and it also has anti-free radical properties.^39^ It was discovered that in addition to reducing cellular energy synthesis and production, AZA may also have anti-free radical activity.^56^ There are many types of cancer cells, and different MCDAs may exist to inhibit or kill their specific cancer cells. SUA is metabolised by body cells and plays a natural role as a media component in the TCA cycle. Hence, it has no negative consequences.^57^ Because SUA proved successful in treating endometrial cancer and had no detrimental or lethal effects on healthy cells, SUA has significant potential to treat cancer.^57^

### 4.4 Nervous regulation drugs

Nowadays, SUA has been shown to prevent mast cell-based systemic anaphylaxis.^58^ Additionally, adsorption of ammonia by SUA can treat hepatic encephalopathy.^59^ These illustrate that SUA plays a great role in the field of neurology (Fig. 3).

According to reports, SUA had hypnotic effects (4000 mg kg^−1^) and protective effects (1180 mg kg^−1^) against high-pressure oxygen convulsion.^60^ Some studies declared that SUA at a dose of 1180 mg kg^−1^ considerably reduced locomotor activity and hyperthermia in mice while also greatly extending the pentobarbital sleeping period.^60^ Additionally, it was shown that SUA prevented rats from experiencing audiogenic seizures and stopped mice from experiencing electroshock seizures, but not those brought on by picrotoxin, strychnine, or semicarbazide. Later, SUA (100–400 mg kg^−1^) suppressed pentylenetetrazol chemical and amygdale ignited seizer in a dose-dependent manner. More in detail, SUA exhibits an anxiolytic-like effect.^60^

Differently, because the addition of GLA alters the redox balance and induces cell death in primary striatal and cortical cultures, it has so far not been used in the treatment of neurological diseases.^61^ In this respect, the activation of neuronal nitric oxide synthase and oxidative stress, as well as the alteration of the mitochondrial membrane potential caused by calcium that GLA causes, is essential for mitochondrial failure.^61^

### 4.5 Medicinal compounds and biomedical material synthesised

So far, in experiments where animals were provided ADA or SUA in the diet, no harmful effects were found.^62^ Consequently, a variety of medicinal compounds are synthesised using SUA as an intermediate (Fig. 3).^2^ For instance, the use of SUA as soluble crystals for the delivery of low-solubility drugs can solve the problem of rapid mismatch during their dissolution and improve the stability and bioavailability of the drug.^5^ Benfurodil, bamethan, chloramphenicol, cibenzoline, deanol, doxylamine, ergotamine, loxapine, metoprolol, oxaflumazine, sumatriptan, and iron (Fe ii) are only a few of the drugs that can be dissolved in SUA.^63^ In this respect, several active pharmaceutical ingredients, including metoprolol, are salt-formed using SUA.^64^ The European Pharmacopoeia and the US FDA both typically recognise ADA and SUA as being safe. As a result, ADA can be used to create various chitosan-based biomaterials, particularly for applications in biomedicine such as tissue engineering, wound dressing, artificial skin, and drug delivery systems, where non-toxicity is a key factor.^65^ Presently, ADA is utilised to make spiramycin (Rovamycine™ injectable form) and piperazine salts (Entacyl™). SA is commonly used as a co-former for the co-crystallisation of pharmaceuticals, just like SUA and ADA.^66^ Clinical d- and l-lactic acids of pharmaceutical grade and SA synthetic copolymers are biocompatible. These copolymers can enhance the delayed release of drugs without resulting in local histopathological problems.^67^ Additionally, SA is frequently employed in the synthesis of medicinal products.^68^

Similarly, GLA is also extensively used in the manufacture of pharmaceuticals, primarily as a component of polymers.^69^ Active pharmaceutical ingredients and GLA (1 : 1 molecular ratio) can form a co-crystal with unique thermal, spectral, X-ray, and solvation properties. In addition to being chemically and physically stable under temperature stress, the co-crystal solid is non-hygroscopic. Comparing the co-crystal to the homomeric crystalline form of the medication boosted the rate of water dissolution by 18 times. The co-crystal enhanced plasma area under curve values by three times at two distinct dose levels, according to single-dose dog exposure tests.^70^

### 4.6 Disease marker

Due to SA's great biocompatibility, stability, and role as a disease marker, it has lately been transformed into new medical devices (Fig. 3).^71^ Diabetic patients excrete much more DAs in their urine than do comparable healthy persons, and DAs excretion declines in the following order: ADA > ethanedioic acid > SA. SA, a stable and readily observable component of DAs, may serve as a marker for the oxidative assault of *cis*-polyunsaturated fatty acids in diabetes.^71^ Additionally, DAs are aberrant in the amniotic fluid of pregnant people at risk for glutaric aciduria type II who have a foetus that is afflicted. The amounts of GLA, ADA, nitrous acid, and SA in the amniotic fluid were all shown to be significantly higher. During pregnancy, this method can accurately identify glutaric aciduria types I and II. It may also be useful in other genetic diseases where DAs accumulate.^72^ Namely, numerous metabolic problems, some of which are linked to the syndromic types of developmental diseases, can be diagnosed by the examination of urine DAs. Hence offering fresh proof that metabolic abnormalities could be one of the underlying causes of this widespread illness, *e.g.* autism spectrum disorders.^21^

### 4.7 Applications of other pharmacological properties

As SA is applied in parenteral nutrition, orthopaedic applications, medication delivery systems, and vaccine production, it is also utilised in a variety of medical procedures (Fig. 3).^73^ SA was suggested as an alternate energy substrate for complete parenteral feeding eight years ago.^74^ By enhancing the activation of oestrogen receptors, SA controls oestrogen signalling, which has sex-dependent benefits for bone, muscle, and adipose tissue.^75^ It is interesting to note that SA can function as new agonists of the capsaicin and transient receptor potential A1 receptors, which, when activated, cause the body to produce more heat and waste more energy.^76^ By lowering the recombinant hepatocyte nuclear factor 4 protein, SA (0.5–1.5 mM) was found to dramatically reduce angiopoietin-like protein 8 expression in human hepatoma HepG2 cells.^77^

Moreover, a formulation consisting of malic, SUA, and citric acids in a ratio of 3 : 2 : 2 inhibits platelet aggregation. It also significantly prevents platelet adhesion without affecting platelet viability.^78^ One year later, the team discovered that the formulation inhibited characteristic molecular on the platelet surface integrin α_IIb_β_3_ expression and extracellular-signal-related kinase activation in platelets, demonstrating a positive antithrombotic effect.^79^

## 5. Existing and potential cosmetological applications of MCDAs

**Fig. 4:**
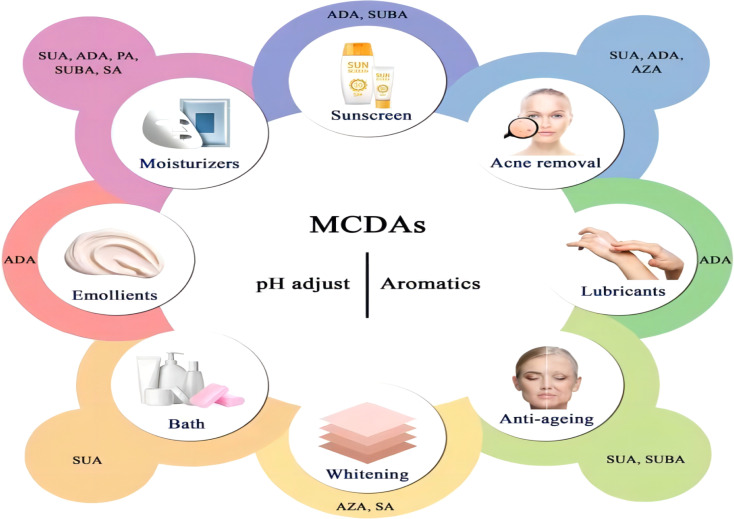
Existing applications of the medium-chain dicarboxylic acids (MCDAs) in cosmetics industries. Abbreviation information: succinic acid (SUA); glutaric acid (GLA); adipic acid (ADA); pimelic acid (PA); suberic acid (SUBA); azelaic acid (AZA); sebacic acid (SA).

Cosmetic products are regulated and categorised differently depending on the nation. In the EU, the term encompasses many other categories, including pharmaceuticals, biocides, and medical devices, and is based on the place of application and intended uses. China further classifies cosmetics into more precise categories, but its classification systems are more comparable to those of the EU. Cosmetics in China are separated into special cosmetics and ordinary cosmetics under the new Cosmetic Supervision and Administration Regulation. Anti-hair loss products, sunscreen, (hyper)pigmentation removal (whitening), hair perming products, hair dyes, and a new category called “cosmetics with new efficacy claim” are considered special cosmetics. The remaining goods are categorised as general cosmetics.^80^ Until present, MCDAs have been widely used in the cosmetics industry (Fig. 4).

### 5.1 General cosmetics

#### 5.1.1 Moisturisers (facial masks)

Hydrogel masks typically have cooling and soothing qualities and are used on sensitive skin.^81^ It is well known that cellulose hydrogels are commonly used in cosmetics and skincare products because of their distinctive properties, such as biocompatibility, high water content, elasticity, and softness. They can also be made from polysaccharides found in naturally occurring plants.^82^ Hydrogels made of networks of hydrophilic polymers are capable of absorbing water towards a thousandfold its dry weight, or between 10 and 20%.^83^ Also, a three-dimensional polymer network, also called a hydrogel, has a high water absorption capacity. Citric acid, SUA, and SA crosslinked cellulose-based hydrogels are biodegradable. They can be utilised as ingredients in cosmetic products. By adjusting the cross-linker concentrations, reaction temperatures, and the use of different cross-linkers, the water absorption of this type of biodegradable hydrogels can be modified.^6^

In general, materials used to make facial masks should have good elasticity and modulus and have a sustained release of the formula as a way of increasing the efficacy of the mask, in addition to preventing the rapid evaporation of the water phase and prolonging the time needed for ingredients to penetrate deeper into the skin to regulate the level of epidermal hydration and to limit the loss of *trans*-epidermal water.^84,85^

The hydrophilic–hydrophobic properties of the mask-forming materials and the steric hindrance of the mask molecular structure both had an impact on the water vapour transfer. The combined actions of water diffusivity and solubility in a polymeric matrix were essential to the water vapour transfer process. Regarding the impact of DAs, composite masks made with ADA had greater water vapour barrier capability than those prepared with SUA. It could be explained by the fact that ADA is more hydrophobic than SUA due to its higher molecular weight and longer carbon chain.^86^ It may also be due to the moles of SUA (comparison with the value 0.0070 of GLA) in an aqueous bulk solution at 298.15 K must be 0.017 to reduce the surface tension by 10% from the value of pure water. Limitedly, SUA (same as GLA) can reduce aqueous surface tension to a maximum of 50 mN m^−1^ at sufficiently high concentrations.^87^ Moreover, the amino groups in the chitosan molecular structure may interact with DAs to produce ionic interactions that keep the chitosan molecules close to one another. Less hydrophilic chemicals may further impede the flow of water vapour through the composite mask matrix because DAs may hold chitosan molecules together.^86^ Hence, DAs may have a strong affinity for protein and chitosan. Indeed, the excellent water retention capacity of chitosan derivatives, *e.g.* succinyl chitosan, makes them ideal for aesthetic applications.^88^

Because of the interaction between the polyvalent organic acids and the terminal amino acids (serine or threonine) of zein molecules, polyvalent organic acids were added to the zein-forming mixture to enhance mechanical qualities. Although both ADA and SUA have crosslinking activity, SUA may have higher crosslinking activity than ADA. SUA-containing composite masks had better tensile strength than ADA-containing composite films. SUA is a better DA than ADA for fabricating stronger composite masks in terms of tensile strength. It is worth mentioning that the antibacterial activity was also regulated by the kinds of acids employed to make the chitosan mask.^86^

To create biocompatible biopolymers with noticeable mechanical properties from natural polymers (chitosan and collagen), PA serves as suitable cross-linkers.^88^ Chitosan and type I collagen can be dissolved with the help of protons from PA. It also engages in ionic contact with both natural polymers. Both natural polymers were dissolved in water when PA was present because of this type of interaction. Scaffolds were made using the resulting solution and put through characterisation tests. The membrane pore structures were evenly distributed throughout the material of scaffolds, which was quite porous. Due to its high porosity, this material may be used in biomedical settings as a matrix for cell growth and absorption sponges, among other things. With an increase in PA concentration up to 0.2%, the biopolymer's mechanical strength increased. Since no harmful substances were used in the processing, the finished product can be used as a cosmetic dressing in addition to being used as a clinical implant or material for treating wounds.^89^

Among these MCDAs, SA and SUBA are commonly used as skin conditioners and moisturisers in skincare products.^90^ SUBA dissociates the natural polymers, such as collagen and chitosan, without the need for acetic acid through the act of proton exchange. It crosslinks and stabilises the natural polymer to create a stable biopolymer material for biomedical purposes. The presence of non-covalent contacts gave the biopolymer product outstanding mechanical and thermal qualities. Furthermore, the fibroblast cells and the biopolymer were compatible. All these findings imply that chitosan and collagen that have been exposed to SUBA may be used as dressing or implant materials for cosmetic purposes.^91^

#### 5.1.2 Acne removal (including blackheads) products

Skin covers the entire human body. Numerous different types of microorganisms live in various body areas. Investigating the effects of different MCDAs on the skin microbiota could lead to the design of more useful cosmetics that work on the skin microbiome. As fungi are generally unable to grow in acidic environments, skin pH adjusters help to prevent the proliferation of these fungi.^92^ Historically, the safety of DAs as they are used in cosmetics has been evaluated by the Cosmetic Ingredient Review Expert Panel.^2^ The bulk of MCDAs serve as pH adjusters or aroma components in cosmetics. Although most of the salts' functions are unknown, sodium succinate is known to have buffering or pH-adjusting properties.^2^ SUA efficiently stops the growth of *Cutibacterium acnes* from growing both *in vitro* and *in vivo* when it is found in the media of *Staphylococcus epidermidis* glycerol fermentation.^7^ Likewise, skin microbiota changes are associated with ageing. Therefore, altering the skin microbiome may result in anti-ageing. Cosmetics that in the principal component analysis showed a positive connection with SUA would be very efficient against *Staphylococcus epidermidis*.^92^

Some of the ingredients that have been used in skin treatments have also been successfully added to cosmetic formulations. The FDA has given AZA the go-ahead to treat rosacea and acne. The topical treatment of mild-to-moderate inflammatory acne vulgaris is advised for a cream containing 20% (w/w) AZA, and the treatment of rosacea is approved for a gel containing 15% AZA.^93^ Only prescriptions are accepted for these medications. (As a point of comparison, it is reported that 0.3% of leave-on formulations and 10% of rinse-off formulations of cosmetics contain AZA).^2^ Equally, in a clinical evaluation, 20% ADA cream alone was 58.3% effective in treating ulcers compared to 27.6% effective with AZA cream. Like AZA, ADA was effective in the treatment of erythema, papules, acne, and pustules. The skin irritation caused by ADA and AZA may be related to a local anaerobic effect, possibly due to their similar mechanism action. However, ADA is usually associated with slightly milder side effects than AZA.^94^ The likely reason for this is that AZA affects the cell membranes of some cells' fragility more than ADA, leading to a decrease in osmotic resistance.^95^ But cells that are sensitive to AZA may have improved diffusion absorption mechanisms or particular DA receptors.^96^ However, in Japan, these two topical preparations have yet to be marketed.^94^

#### 5.1.3 Other general cosmetics

Although some of the MCDAs have been shown to have an antibacterial effect, the most reported uses for the MCDAs and their salts are for disodium succinate. The acid with the highest use concentration is SUA, which can be used at a concentration of up to 26% in bath products before being diluted. With dermal contact exposure, disodium succinate had the highest leave-on concentration at 0.4%.^2^

The word “emollient” is frequently used interchangeably with moisturiser ingredients, including occlusive and humectant substances, as well as real emollients. Emollients have moisturising capabilities in addition to being strongly tied to the sensory qualities of cosmetics.^97^ Until now, ADA, only one of the MCDAs, is used in the manufacturing of lubricants and emollients in the cosmetic industry.^62^ Further, in recent years, the esters of SA have also been employed as skin pH adjusters and plasticisers in cosmetic items.^98^

The universality of the succinate/succinate receptor 1 system's angiogenic effects appeared in several tissues. It was discovered that Na succinate 1.6% had a direct impact on the level of synthesis of endogenous growth factors that determine the speed of tissue regeneration.^99^ Furthermore, it was demonstrated that it is possible to affect chronic inflammatory processes directly, and it was discovered that using a Na succinate 1.6% preparation, it is possible to deliberately start the polarisation of skin macrophages from the pro-inflammatory M1 phenotype into the anti-inflammatory M2 phenotype. Because it is well known that the process of chronic inflammation is one of the fundamentals in the pathogenesis of ageing, this discovery creates enormous opportunities for doctors of various specialities, including dermatologists and plastic surgeons, in the planned initiation of regeneration, repair, and rejuvenation processes, as well as the blocking of the willow processes in the tissues associated with the presence of chronic inflammation.^99^

### 5.2 Special cosmetics

#### 5.2.1 (Hyper)pigmentation removing (whitening) products

It has been demonstrated that AZA and other saturated DAs (C9–C12) are competitive inhibitors of membrane-associated thioredoxin reductase and tyrosinase.^8^ AZA reversibly inhibits tyrosinase, and melanin biosynthesis has been directly hampered. It has been utilised in hyperpigmentation treatment (lentigo maligna and melasma) due to its depigmenting properties and minimal toxicity. It has been discovered that AZA acts as a bacteriostatic agent by blocking DNA synthesis in some of the bacteria linked to acne vulgaris. In contrast, other bacteria have proven resistant to this DA's inhibition. Since the two terminal carboxylate moieties of AZA and other active saturated DAs contain the sole chemically reactive groups. The membrane-associated thioredoxin reductase, which is found on the surface of keratinocytes, melanocytes, and melanoma cells, as well as purified enzymes from *Escherichia coli* and human metastatic melanotic melanoma cells, is reversibly inhibited by AZA and other saturated DAs. An NADPH/TR/thioredoxin/tyrosinase feedback system has been established to control melanin biosynthesis and diminish free radicals at the surface of the human epidermis. The reversibility of thioredoxin reductase inhibition by AZA is one of its most significant features. After the medicine is gone, DNA synthesis and pigmentation return to normal. Using 14C-labelled AZA and evidence of the production of a putative thioester on the thiolate active site of this enzyme, a cause for this reversibility has been shown.^8^

#### 5.2.2 Sunscreen

In hairless mice, supplementation with SUBA prevented ultraviolet B-induced skin photoaging. SUBA taken orally prevented the development of wrinkles, dry skin, and increased epidermal thickness caused by ultraviolet B exposure. SUBA works by increasing the expression of molecules involved in the general outline of the TGF-β receptor/smad signal transduction pathway and deactivating the mitogen-activated protein kinase/activator protein-1 pathway.^100^ It was demonstrated that dietary SUBA shields the skin of hairless mice against ultraviolet B-induced ageing by boosting the levels of collagen and genes that produce it, including collagen type I alpha 1 chain.^100^ In the back skin of mice, SUBA taken orally decreased the expression of matrix metallopeptidase-1, -3, and -9 while increasing the expression of collagen and hyaluronic acid.^100^

SUBA's molecular target is mostly unknown, but certain investigations have found evidence that the DA series, which includes SUBA, may interact with the olfactory receptors in mammalian cells. In dermal fibroblasts that have been exposed to UV radiation, the activation of olfactory receptors 10A3 (OR10A3) by SUBA enhances the protein kinase A system/protein kinase B signalling pathway, which helps to promote collagen synthesis. A therapeutic target for skin anti-ageing may be OR10A3.^101^ However, studies on the topical use of SUBA in cosmetics have not been reported.

It is important to measure the quantity of ultraviolet-absorbing compounds that get into the body through the skin. The nature of the vehicle may alter the stratum corneum's characteristics (*e.g.* enhanced hydration), which could affect the active components' penetration profile.^102^ Based on skin-stripping tests on humans, the penetration of octyl methoxycinnamate and benzophenone-3 into live skin was reduced when 25% ADA/diethylene glycol/glycerin cross-polymer was added to sunscreen solutions, suggesting that the barrier function of the stratum corneum is enhanced, limiting and reducing the rate at which compounds penetrate the skin.^102^

### 5.3 Further inspiration of the pharmacological properties of MCDAs for cosmetic development

Since the skin organ includes all the body's primary support systems, including blood, innervation, and muscle, as well as its immuno-competence, psycho-emotion responsiveness, ultraviolet radiation sensitivity, and endocrine functions, it truly is a biological cosmos. Together, they play a role in maintaining the homeostasis of the skin and its appendages, which is critical for maintaining the overall mammalian body's homeostasis.^103^

**Fig. 5:**
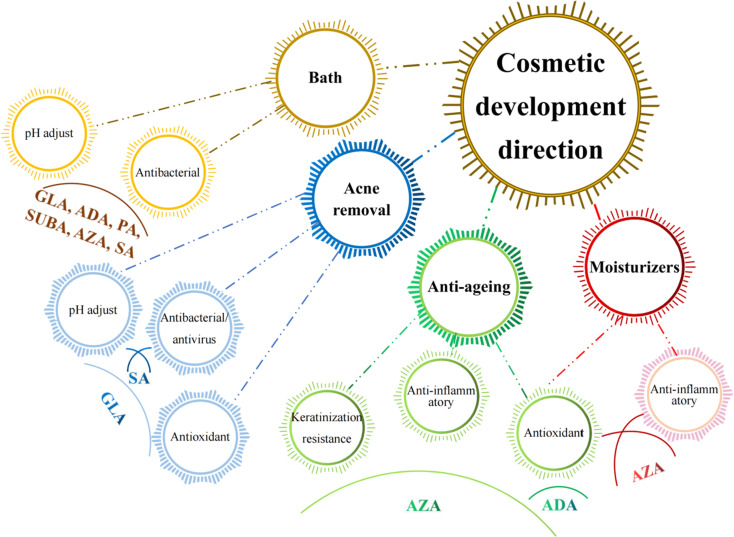
Potential applications of the medium-chain dicarboxylic acids in cosmetics industries according to their pharmacological properties. Abbreviation information: glutaric acid (GLA); adipic acid (ADA); pimelic acid (PA); suberic acid (SUBA); azelaic acid (AZA); sebacic acid (SA).

It is known that a variety of recognised aetiologies can cause skin injury. The occurrence of moisture-associated skin damage (MASD) may depend on several factors, including the chemical composition of the moisture source, its pH, mechanical factors like friction, and the presence of potentially pathogenic bacteria. Dermatitis is a common presenting form of MASD.^104^ Even though MASD has been linked to many bacterial species, different species of MCDAs inhibit both bacteria and viruses to varying degrees, as mentioned above. For instance, SA, SUA, and AZA are used in non-pharmaceutical applications to inhibit the growth of *Staphylococcus aureus*,^105–107^*Escherichia coli*^105,106,108^ and *Botrytis cinerea*^109,110^ as well as other strains of bacteria. These inhibition effects would explain why SUA, ADA, and AZA can be developed as moisturisers or acne removal products. The difference is that, to our knowledge, there are no reports that SA and GLA can be developed as acne removal products and AZA as moisturisers (Fig. 5).

Healthy skin has a pH range of 5.5 to 5.9. This somewhat acidic layer performs many crucial tasks, one of which is to suppress coliform bacteria, which prefer an alkaline environment. When the skin's surface is blocked, more carbon dioxide is produced, the pH becomes more alkaline, and the relative humidity rises. It becomes clear how crucial the acid mantle is to the moisture barrier. Raising the pH of the skin prevents freshly produced lipids from assuming their distinctive layered structure (lamellar), reduces corneocyte adhesion, and enhances corneocyte lysis.^111^ However, at present, except for SUA, other MCDAs have not been applied to bath products to perform daily care of skin pH.

A gradual, proportional loss in the effectiveness of the skin's moisture barrier function is linked to ageing.^112^ The skin ages both internally (chronologically), which is influenced by genetic and hormonal factors, and externally, which is brought on by environmental factors such as UV radiation, smoking, nutrition, chemicals, trauma, *etc.*^113^ Since multiple factors often combine to influence the ageing process, different species of MCDAs have the potential to be developed into anti-ageing products and sunscreens.

## 6. Conclusions

The review that has been provided demonstrates how appealing MCDAs are as a subject for many scientific investigations. Current pharmacological research has validated the benefits of MCDAs for health. It also compiles information from multiple research studies to lay a foundation for the seven compounds' (SA, SUA, AZA, GLA, ADA, SUBA, and PA) potential application in cosmetics. Indeed, the pharmacological properties of MCDAs extend beyond their traditional medical applications. Their bacteriostatic activity, for example, holds promise for various cosmetic applications, potentially informing the development of skincare products aimed at addressing bacterial skin conditions. However, it is important to note that the physiological effects of these acids may vary depending on their route of administration and concentration in the body. Research into the mechanisms of action, optimal concentrations, and compatibility of these acids with other substances is essential for their successful implementation in cosmetics and pharmaceuticals. Understanding how these acids interact with the skin and other components of cosmetic formulations can help optimise their efficacy and safety. Further mechanistic studies and exploration of compatibility and stability are needed to fully harness the potential of MCDAs in the cosmetic and pharmaceutical industries. Continued research in this area holds promise for the development of innovative products with diverse applications.

## Abbreviations

ADA: Adipic acid
AZA: Azelaic acid
DAs: Dicarboxylic acids
GLA: Glutaric acid
MASD: Moisture-associated skin damage
MCDAs: Medium-chain dicarboxylic acids
Olfr544: Olfactory receptor 544
PA: Pimelic acid
SA: Sebacic acids
SUA: Succinic acid
SUBA: Suberic acid

## Author contributions

Zhengrui Liao: conceptualization, validation, visualization, writing – draft, and editing. Yu-Kee Yeoh: visualization and conceptualization. Thaigarajan Parumasivam: investigation, methodology and data curation. Wee Yin Koh: editing. Mohammad Alrosan: writing – correction and review. Muhammad H. Alu'datt: writing – correction and review. Thuan-Chew Tan: writing – review and editing.

## Conflicts of interest

There are no conflicts of interest.

## Supplementary Material
